# They didn’t cover this in lecture: The formation and dissolution of the patient-provider relationship

**DOI:** 10.1007/s40037-018-0426-9

**Published:** 2018-04-24

**Authors:** Kathryn S. Czepiel

**Affiliations:** Medical School, University of Massachusetts, Worcester, MA USA

**Keywords:** Adolescent health, Medical education, Relationships in medicine

## The story

When I returned to the delivery room post-delivery, I was met by an eerie quiet. No cries of a baby or the lulls of family in conversation. Instead, my eyes were met by a chaotic scene of haphazardly thrown hospital blankets and blood-tinged gowns left abandoned in the wake of the delivery. My mind raced to keep pace with the rapid turn of events. The baby was safe and well in the newborn nursery, but her mother, Danielle, lay far from her side.

I was fatefully paired to work with Danielle through a longitudinal elective course offered to third-year students at my medical school. This elective bridges the fields of obstetrics and paediatrics, as it provides medical students the opportunity to follow an expectant mother throughout her late pregnancy and then work with her child as he or she establishes care in a paediatric office. Danielle is a local high school student whom I met during her 34th week of pregnancy—a pregnancy that was unplanned, but wanted. My experience was rather unique from that of my classmates because Danielle was a paediatric patient herself.

From the start of our patient-provider relationship, Danielle was well-informed and engaged in her medical treatment. She asked appropriate questions and was 100% dedicated to her unborn daughter. It quickly became evident that I was privy to a major life event for Danielle, as I sat by her side for all obstetrical appointments and communicated with her regularly in between doctor visits. In the days leading up to her expected due date, Danielle and I convened in a restless waiting room to discuss birthing plans. These plans included a host of preferences that were important to Danielle in providing her and her daughter a safe birthing experience. Inspired by Internet blogs and iPhone pregnancy apps, she spoke of her plans for her upcoming delivery: anaesthesia options, birthing positions and newborn care instructions.

On a Friday night in September I received the message that I had been waiting for: Danielle was in labour. I remember driving to the hospital with jittery excitement. This was the moment she (and I) had been thinking about for weeks.

Danielle’s delivery went well—until it didn’t. After Penny was born, Danielle’s uterus failed to contract, causing her to lose nearly two litres of blood within minutes. As a resident physician worked tirelessly to manually contract Danielle’s uterus, other teams of physicians flooded the tightly-packed room. There was no time for enacting any birth plans. I recalled Danielle’s hopes for this delivery and how its reality so quickly changed. As Danielle was whisked away to the operating room, I stood by Penny’s side. I followed her to the nursery with her now-shaken father and grandmother. Despite feeling downhearted, we shared a common goal: to implement what parts we could of Danielle’s birth plan.

Eventually, Danielle’s bleeding was controlled with a Bakri balloon tamponade, an intrauterine catheter that is placed surgically. I visited her late that night as she lay in the surgical intensive care unit, still asleep, intubated and alone. No one could have predicted this outcome. I realized then that my relationship with Danielle, and now Penny, was more than just a school assignment.

In the days following the delivery, I struggled not knowing how to appropriately involve myself during this difficult time, especially as a student. I chose to respect Danielle by giving her time and space to recover surrounded by loved ones and a full team of Ob/gyn staff, though my thoughts stayed with her in the days following the delivery. I opted for check-ins via phone and text to let Danielle know that I was there if she needed anything. It was almost a week later at Penny’s intake appointment with her paediatrician that I first had the chance to see Danielle again. A sense of welcomed relief washed over me as I watched a recovering Danielle smile and rock Penny in her arms. The paediatrician confirmed our thoughts: birth plan or no birth plan, Penny was perfect.

## Surprising outcomes

In the weeks following the delivery, I started my assigned psychiatry clerkship as a student on an adolescent unit. Within days, I was again thrown into close patient-provider relationships with new, vulnerable patients. I was again humbled by the teenagers’ willingness to share their deepest insecurities and anxieties with me. Before I knew it, my five weeks were up and it was my time to start a new assignment on yet another clerkship. I worked very consciously to bring some comfort and closure to the relationships I had developed on this rotation—scheduling face-to-face meetings with each of my patients—acknowledging our time together and my transitioning away from their care. As I said my goodbyes, I was struck by the seemingly frequent forming and dissolving of relationships that I had experienced while caring for patients in the short bursts of time that are the structure of my medical training. It made me appreciate the magnitude and potential impact of those relationships—for both the patient and for me, the provider in training. I couldn’t help but think of my relationship with Danielle and Penny. After going through so much with Danielle, communicating on a regular basis and being a part of such a private and special life event, I struggled to see a way to appropriately end our relationship. A pre-scheduled elective end date did not seem sufficient.

## Lessons learned

Looking back, I know now that the patient-provider relationship with Danielle and Penny ended that day in the paediatric office. That’s largely because the elective’s ‘assignment’ was complete. I think it is natural for medical students and other healthcare professionals to want to utilize interpersonal skills to enter into meaningful relationships with others. In my experience, one of the greatest attributes of medical professionals is the ability to connect with others, often in times of difficulty and stress. These relationships make the monotonous hours spent studying and writing notes worthwhile and remind us why we chose such a profession. Without these human interactions, much of my time and effort would feel futile. However, we must balance this with the need to maintain relationships with patients that are professional, not personal. I had not considered the impact of developing and then ending these relationships. No matter how brief, all relationships carry meaning and can greatly affect those who are involved, especially given the fact that these interactions are often initiated around a major life event, like childbirth.

I often think about how fortunate I was to be paired with Danielle. She taught me greatly about what it is like to experience the healthcare system as a young mother. She also let me be a part of her very intimate and personal story. I recognize that what we shared is rare, and I certainly cannot expect that every future patient-provider relationship that I enter into will evolve so gracefully and with such meaning. Thanks to her, I will remain conscious and careful around the development and dissolution of relationships with those I am fortunate enough to care for as a medical student and future physician.

## Moral of the story

I think it is important to stay forever mindful of how fortunate we are to be a part of our patients’ very personal experiences. In addition, the relationships that we form on a regular basis within medicine can be impactful for both patient and provider and thus we must be careful, conscious and deliberate in how we develop and dissolve such relationships.

